# Impact of the COVID-19 Pandemic on the Management of Acute Colonic Diverticulitis: A Retrospective Observational Study

**DOI:** 10.7759/cureus.85427

**Published:** 2025-06-05

**Authors:** Manuela Mastronardi, Aurora Bertini, Margherita Sandano, Paola Germani, Marina Troian, Nicolò de Manzini, Alan Biloslavo

**Affiliations:** 1 Department of Medicine, Surgery and Health Sciences, University of Trieste, Trieste, ITA; 2 Department of Surgery, San Carlo Borromeo Hospital, Azienda Socio Sanitaria Territoriale Santi Paolo e Carlo, Milan, ITA; 3 General Surgery Unit, Cattinara University Hospital, Azienda sanitaria universitaria Giuliano Isontina, Trieste, ITA; 4 Thoracic Surgery Unit, Cattinara University Hospital, Azienda sanitaria universitaria Giuliano Isontina, Trieste, ITA

**Keywords:** acute diverticulitis, acute diverticulitis admission, conservative vs surgical management, covid-19, digestive surgery, outpatient clinic

## Abstract

Introduction

Acute colonic diverticulitis (ACD) is a frequent gastrointestinal disease, with its rising incidence and hospitalizations contributing to increased healthcare costs. The COVID-19 pandemic significantly impacted healthcare delivery, leading to shifts in hospital admissions and management strategies for various conditions, including ACD. This study aims to assess the changes in hospital admission rates for ACD before and during the COVID-19 pandemic. Secondary outcomes include the differences in patient demographics, management approaches, and follow-up data between the two periods.

Methods

A retrospective observational study was conducted on patients admitted with ACD between January 1, 2017, and May 5, 2023. Patients were divided into two groups: the pre-COVID group (PCG, January 2017 to March 2020) and the COVID group (CG, March 2020 to May 2023). Data collected included patient characteristics, laboratory findings, disease severity, imaging, treatment strategies, and the 90-day follow-up outcomes. Statistical analyses were used to compare the two groups.

Results

A total of 428 patients were included in the study (235 in PCG, 193 in CG), with a 17.87% decline in hospital admissions for ACD during the pandemic. While the demographic characteristics were largely comparable, previous abdominal surgery was more frequent in PCG (p=0.006). Management strategies shifted, with the increased use of radiological drainage in CG (p=0.034), though surgical intervention rates remained stable. CRP levels were significantly higher in CG (p=0.010), suggesting a possible delay in care-seeking.

Conclusion

The COVID-19 pandemic led to a reduction in ACD-related hospital admissions, with an increased use of radiologic drainage. Overall care quality remained consistent, emphasizing the healthcare system's resilience during crises.

## Introduction

Acute colonic diverticulitis (ACD) is one of the most common gastrointestinal disorders in Western countries, with an estimated lifetime risk of 10-25% in patients with diverticulosis [1,2]. Predominantly affecting the left side of the colon, its prevalence has been increasing markedly, with an estimated rise of up to 132% from 1980 to 2007, especially among individuals aged 40-49 years [3-6]. Italy is not an exception, as Amato et al. [7] documented an increasing rate of hospitalization for ACD from 2008 to 2015, especially for patients younger than 60 years. Unsurprisingly, the rise in incidence and hospitalization rates has been subsequently characterized by a multiplication of ACD-related healthcare costs [8,9].

The COVID-19 pandemic profoundly impacted healthcare systems worldwide, altering the patterns of hospital admissions and the management of several conditions, including ACD [10-13]. There was a noticeable decline in the number of patients seeking care in emergency departments, particularly for thoracic-abdominal issues, accompanied by a rise in cases of advanced and severe forms presenting in a critical condition [10]. Overall, the pandemic catalyzed a shift in the management of ACD toward less invasive and more conservative approaches to reduce hospital admissions and resource utilization [10]. Many patients were managed on an outpatient basis with minimal intervention, including telephonic follow-up to monitor their progress [10,14]. For cases involving abscess formation, percutaneous drainage became the treatment of choice, reducing the need for emergency surgical interventions [15-17]. Surgical procedures were largely reserved for extreme complications such as diffuse peritonitis or sepsis [14].

Although these changes were implemented out of necessity during the peak of the pandemic, they represent an important shift in ACD management that warrants careful evaluation to guide future practice. Given the strain on hospital resources and changes in healthcare-seeking behavior during the pandemic, it is important to understand how these factors influenced the incidence and management of ACD.

On this basis, the present study aims to assess the changes in hospital admission rates for ACD before and during the COVID-19 pandemic. Secondary outcomes included comparisons between the two groups regarding patient demographics, management strategies, and follow-up data.

## Materials and methods

This retrospective observational cohort study was based on the electronic data of patients with ACD admitted to our center between January 1, 2017 and May 5, 2023.

All patients aged over 18 years who were diagnosed with ACD were included in the study. Given the retrospective nature of the study, patients were identified through their electronic medical records. We selected those whose hospital admission was primarily coded with the International Classification of Diseases (ICD) 9 diagnosis for ACD. Only patients with ACD listed as the primary diagnosis for that specific hospital admission were included. The exclusion criteria were diverticular stenosis causing bowel obstruction and incomplete medical records.

The study population was divided into two groups based on the time of admission: (a) pre-COVID group or PCG, which included patients admitted from January 1, 2017 to March 9, 2020 (38 months), and (b) COVID group or CG, with admissions between March 10, 2020 and May 5, 2023 (38 months).

The timeframes were selected to provide an equal duration of 38 months for both the PCG and CG, enhancing the comparability of data. The cut-off date of March 10, 2020, was chosen to coincide with the onset of the first lockdown and the significant pandemic-related changes in healthcare management [18].

The patient data collected included age, sex, body mass index (BMI; kg/m^2^), Charlson Comorbidity Index (CCI), American Society of Anesthesiologists Physical Status Classification (ASA) score, previous abdominal surgery, known diverticular disease, inflammatory markers (white blood cell count or WBC count; units/μl), C-reactive protein (CRP; mg/dL), disease severity based on the Hinchey classification according to Wasvary [19], diagnostic imaging (contrast-enhanced computed tomography or CE-CT, abdominal ultrasound), and treatment strategies (non-operative management or NOM, percutaneous drainage, and surgical intervention).

NOM consisted of hydration, analgesic therapy, and antibiotic therapy when indicated. Percutaneous drainage was performed for abdominal collections exceeding 4 cm [2].

Surgical interventions were classified based on the approach, either laparoscopic or open surgery, and the specific procedure, such as sigmoid resection with primary anastomosis (with or without protective ileostomy) or the Hartmann procedure.

Moreover, data on the use of antibiotic therapy, either oral or intravenous, and the total length of hospital stay (LOS) were collected.

Patients were followed for 90 days after their hospital admission. Follow-up data included completion of visits in the colorectal or emergency outpatient offices (started in January 2021), completion of colonoscopy within two months of resolution, whether they were planned for elective sigmoidectomy or not, and readmission rates.

Laboratory tests, including WBC and CRP, were processed at the hospital’s central laboratory using consistent instrumentation and standard operating procedures throughout the study period.

Diagnostic imaging, including CE-CT scans and ultrasound, was performed using the hospital’s routine protocols. All imaging and laboratory evaluations followed institutional standards, ensuring consistency across the two study periods.

During the pre-pandemic period, patients with ACD were admitted via different routes, including direct emergency room (ER) visits and outpatient clinic referrals. However, during the pandemic, outpatient clinics were suspended due to the emergency, leaving the ER as the only entry point for hospital admissions.

Statistical analysis

Considering that both periods analyzed were 38 months long, we calculated the percentage change in the hospital presentation rate for ACD using the following formula [20]:

(Number of admissions in CG - Number of admissions in PCG)/Number of admissions in PCG * 100.

Continuous variables were summarized as means with standard deviations (SD) or medians with ranges depending on the distribution. The Shapiro-Wilk test was used to assess the normal distribution of quantitative variables. Only the variable age showed a normal distribution. The variables BMI, CCI, WBC, CRP, and LOS were continuous but not normally distributed. Categorical variables (sex, ASA, previous abdominal surgery, known diverticular disease, ACD grade, diagnostic imaging, management type, antibiotic therapy, colonoscopy, NOM failure, and type of surgery) were expressed as frequencies and percentages. Comparisons between the pre-COVID-19 and the COVID-19 periods were made using appropriate statistical tests. For continuous variables, either independent t-tests (only for normally distributed variables, i.e. age) or Mann-Whitney U tests (for non-normally distributed variables) were used, while categorical variables were compared using chi-square or Fisher’s exact tests. 

A multinomial logistic regression was performed to identify which factors among age, sex, BMI, WBC, CRP, the period of admission (before or during the pandemic), and disease grade according to Hinchey classification, influenced the treatment strategy (NOM, NOM with radiologic drainage, and surgery).

A p-value of <0.05 was considered statistically significant. Data were analyzed using IBM SPSS Statistics for Windows, Version 23 (Released 2015; IBM Corp., Armonk, New York, United States). 

Ethical considerations

Due to its retrospective and observational design, an approval by the local ethics committee was waived. All data were handled under the Declaration of Helsinki.

## Results

Between January 1, 2017 and May 5, 2023, 428 patients with a diagnosis of ACD met the inclusion and exclusion criteria and were admitted to our hospital. Of these, 235 were included in PCG and 193 in CG, representing a 17.87% reduction in ACD presentations during the pandemic period.

The two groups were similar in demographic characteristics, except for a higher rate of previous abdominal surgery in PCG. Laboratory values showed comparable WBC counts across both groups, while CRP levels were elevated in CG, suggesting a more inflammatory presentation. Disease severity, as measured by ACD grade, did not differ significantly between the groups. No patients in CG had an active COVID-19 infection at the time of admission (Table 1).

**Table 1:** Comparison of the demographics, clinical characteristics, and disease severity between the pre-COVID and COVID groups ^°^chi-squared test; ^§^t-test; ^^^Mann-Whitney test; PCG: pre-COVID group; CG: COVID group; BMI: body mass index; F: female; M: male; CCI: Charlson Comorbidity Index; ASA: America Society of Anaesthesiologists; WBC: white blood cells; CRP: C-reactive protein; AD: acute diverticulitis. Nominal data are expressed in number (%), continuous quantitative variables in mean±standard deviation; non-continuous variables in median and range.

	PCG (n=235)	CG (n=193)	P-value	Statistic value
Age	68.5±14.7	69.7±16.1	0.428	3.720^§^
Sex			0.763	0.124°
-Female	85 (36.2)	73 (37.8)		
-Male	150 (63.8)	120 (62.2)		
BMI	24.5 (18.7-36.3)	25.4 (18.5-35.2)	0.219	-1.065^
CCI	3 (0-10)	3 (0-9)	0.309	15.095°
ASA			0.689	1.509°
-1	90 (38.3)	72 (37.3)		
-2	92 (39.1)	80 (41.5)		
-3	50 (21.3)	36 (18.7)		
-4	3 (1.3)	5 (2.6)		
Previous abdominal surgery	138 (59.0)	86 (45.5)	0.006	7.617°
Known diverticular disease	79 (33.6)	66 (34.2)	0.919	0.016°
WBC (X 10^3^ UI/µL)	12.6 (4.6-24.6)	13.0 (1.4-256.0)	0.721	-0.357^
CRP (mg/dL)	71.2 (0.2-420.0)	107.0 (0.8-445.5)	0.010	-2.589^
AD grade according to Hinchey			0.693	2.209°
-0	67 (28.5)	64 (33.2)		
-1	131 (55.7)	97 (50.3)		
-2	15 (6.4)	10 (5.2)		
-3	19 (8.1)	18 (9.3)		
-4	3 (1.3)	4 (2.1)		

The use of CT scans was consistent between the two groups. However, differences emerged in the treatment strategies (Table 2).

**Table 2:** Comparison of the diagnostic imaging, management strategies, and outcomes between the pre-COVID and COVID groups ^°^chi-squared test; ^^^Mann-Whitney test; PCG: pre-COVID group; CG: COVID group; CT: computer tomography; US: ultrasound; MRI: magnetic resonance imaging; NOM: non-operative management; HR: Hartmann Resection; Lps: laparoscopic; LAR: low anterior resection; DCS: damage control surgery; LOS: length of stay. Nominal data are expressed in number (%), continuous quantitative variables in mean±standard deviation; non-continuous variables in median and range.

	PCG (n=235)	CG (n=193)	P-value	Statistic values
Diagnostic imaging			0.239	4.334°
None	15 (6.4)	6 (3.1)		
CT scan	208 (88.5)	179 (92.7)		
US	4 (1.7)	5 (2.6)		
MRI	8 (3.4)	3 (1.6)		
Management			0.034	6.738°
NOM	206 (87.7)	154 (79.9)		
NOM + radiological drainage	6 (2.6)	14 (7.3)		
Surgery	23 (9.8)	25 (13.0)		
Antibiotic therapy	232 (89.7)	193 (100)	0.255	2.481°
Colonoscopy	16 (6.8)	20 (10.4)	0.221	1.738°
NOM failure	12 (5.1)	9 (4.7)	0.169	3.726°
Type of surgery			0.905	3.058°
Open HR	15 (46.9)	11 (39.3)		
Lps HR	1 (3.1)	1 (3.6)		
Open LAR	8 (25.0)	6 (24.1)		
Lps LAR	6 (18.8)	7 (25.0)		
Stoma	0	1 (3.6)		
DCS	2 (6.3)	2 (7.7)		
LOS (days)	6.0 (0-40)	6.0 (1-60)	0.066	-1.838^

NOM was more frequently employed in PCG, while NOM combined with radiological drainage was more common in CG. Surgical intervention was slightly more frequent in CG, though the overall rates remained low in both groups. No substantial differences were found between the groups in terms of antibiotic use, colonoscopy rates, NOM failure, types of surgical procedures, or LOS.

Follow-up data, including recurrence and re-intervention rates, showed no significant differences between the two groups (Table 3).

**Table 3:** Comparison of the follow-up outcomes between the pre-COVID and COVID groups ^°^chi-squared test; PCG: pre-COVID group; CG: COVID group; NOM: non-operative management; LDG: low-grade dysplasia; HDG: high-grade dysplasia. Nominal data are expressed in number (%), continuous quantitative variables in mean±standard deviation; non-continuous variables in median and range.

	PCG (n=235)	CG (n=193)	P-value	Statistic value
Follow-up in emergency surgery outpatient clinic (from January 2021)	-	40 (20.7)	-	
Follow-up in the colorectal outpatient clinic	141 (60.0)	111 (57.5)	0.623	0.271°
Follow-up colonoscopy	82 (34.9)	59 (30.6)	0.354	0.897°
Endoscopic findings			0.426	3.551°
-Diverticula	71 (86.6)	46 (78.0)		
-Cancer	1 (1.2)	1 (1.7)		
-LDG adenoma	8 (9.8)	7 (11.9)		
-HDG adenoma	1 (1.2)	4 (6.8)		
-Other	1 (1.2)	1 (1.7)		
90-day readmission	18 (7.7)	8 (4.1)	0.156	2.294°
Elective surgery	17 (8.0)	13 (7.7)	1.000	0.010°

The multinomial logistic regression analysis (Table 4) indicated that patients with a higher BMI were more likely to be managed conservatively.

**Table 4:** Multivariate analysis NOM: non-operative management; BMI: body mass index; WBC: white blood cells; CRP: C-reactive protein; CI: confidence interval. The analysis was conducted to identify if the treatment strategy has been influenced by factors such as age, sex, BMI, and inflammatory parameters (WBC, CRP), time of admission (before or during the pandemic), and grade of the disease according to the Hinchey classification.

Predictor	Coefficent	Standard error	Wald	p-value	Odds ratio	95% CI
NOM - Intercept	3.868	3.18	1.479	0.224		
Age	-0.018	0.022	0.655	0.418	0.982	0.941-1.025
Sex	-0.116	0.672	0.03	0.863	0.89	0.239-3.32
BMI	0.208	0.104	3.981	0.046	1.231	1.004-1.51
WBC	0.016	0.017	0.916	0.338	1.016	0.983-1.051
CRP	-0.001	0.003	0.028	0.868	0.999	0.993-1.006
Pre/during COVID	-1.056	0.614	2.956	0.086	0.348	0.104-1.159
Hinchey Grade	-2.834	0.418	45.974	<0.001	0.059	0.026-0.133
NOM + drainage - Intercept	1.127	3.826	0.087	0.768		
Age	0.022	0.027	0.631	0.427	1.022	0.968-1.079
Sex	-0.84	0.827	1.031	0.310	0.432	0.085-2.184
BMI	-0.048	0.123	0.151	0.698	0.953	0.749-1.213
WBC	0.016	0.021	0.612	0.434	1.016	0.976-1.058
CRP	0.008	0.004	3.968	0.046	1.008	1.0-1.015
Pre/during COVID	0.458	0.779	0.346	0.556	1.582	0.343-7.287
Hinchey grade	-1.59	0.456	12.161	<0.001	0.204	0.083-0.498
Surgery - Reference	-	-	-	-	-	-

In contrast, those with more severe disease, as indicated by a higher Hinchey grade, were more often treated surgically. Elevated CRP levels were associated with the use of NOM combined with drainage. Other variables, including age, sex, WBC count, and the time of admission (pre- or during COVID-19), were not independently associated with treatment choice.

## Discussion

During the COVID-19 pandemic, the management of ACD likely underwent significant changes due to the widespread disruptions in healthcare systems.

This study provides insight into the impact of the COVID-19 pandemic on hospital admissions and management strategies at a single tertiary care center. Consistent with previous reports, our findings demonstrated a reduction in the hospital presentation rate for ACD during the pandemic, with a decrease of 17.87% compared to the pre-COVID period. It is important to note that our hospital did not implement ER diversion protocols during the COVID-19 pandemic, ensuring continuous access to emergency care for patients with ACD. However, despite this, we observed a 17.87% decline in hospital admissions, which may be attributed to patient hesitancy in seeking care due to pandemic-related concerns. This is in line with prior studies reporting a 21.6% and 19.3% drop in hospitalizations during the first and second waves of the COVID-19 pandemic, predominantly affecting admissions for uncomplicated diverticulitis, alongside an 11.6% and 16.8% increase in hospitalizations for complicated cases [12]. Several studies similarly observed a decline in ACD diagnosis during the same period [21-23].

This decline is likely multifactorial, including reduced healthcare-seeking behavior due to fear of COVID-19 exposure and changes in healthcare system priorities during the pandemic. These results align with broader trends observed for other non-COVID-19 medical conditions during the same period [24-26].

Despite the decrease in hospital admissions, patient demographics, clinical severity, and laboratory parameters remained largely comparable between the two periods in our study. However, we observed a significant difference in the rate of previous abdominal surgeries, which was higher in PCG. This may suggest a different profile of disease chronicity or recurrence, with a more chronic or recurrent disease pattern presenting before the pandemic, whereas the pandemic period may have resulted in delayed presentations or patients seeking care only when the symptoms were severe.

Despite similar Hinchey grades, management strategies for ACD differed significantly between the two periods. NOM was the primary approach in both groups but was used more frequently in the pre-COVID period (87.7% vs. 79.9%). Conversely, the use of radiological drainage increased during the pandemic. This change may have been driven by efforts to reduce hospital resource utilization and avoid surgical risks during the time of heightened strain on healthcare systems. While surgical intervention rates were slightly higher in CG, the difference was not statistically significant, suggesting that the severity of cases requiring surgery remained relatively stable. This is in line with the current literature, which shows a prevalence of NOM whenever possible [10,14,26].

CRP levels were significantly higher in CG, indicating a potential delay in seeking care during the pandemic, leading to more pronounced inflammatory responses at admission. Elevated CRP is a reliable marker of disease severity in ACD and may influence clinical decision-making [27].

This finding supports the hypothesis that patients may have delayed seeking medical care until their symptoms became severe, driven by the fear of contracting an infection while visiting the hospital [28]. Additionally, outpatient clinics were not operational during the pandemic, making the ER the only access point for hospital admission. This structural change in healthcare access may have further contributed to delays in presentation, as patients no longer had the option of earlier outpatient evaluation and management.

No significant differences were observed in the type of surgical procedures performed, LOS, or follow-up outcomes, suggesting that despite changes in access and care pathways, the overall consistency of care delivery remained unchanged. Furthermore, none of the patients in CG were documented as having active COVID-19 infection during their admission, suggesting effective triaging and infection control measures.

Our findings suggest that clinical severity and inflammatory status played a central role in guiding treatment decisions for patients with ACD. Specifically, higher Hinchey grades were strongly associated with lower odds of receiving NOM or NOM with drainage, emphasizing that surgical intervention remains the preferred approach in the more advanced disease stages. Interestingly, patients with a higher BMI were more likely to receive conservative treatment, which may reflect a tendency to avoid surgical risks in individuals with increased perioperative vulnerability. This is in line with previous studies indicating that obesity is associated with increased perioperative risk and higher rates of postoperative complications in patients undergoing colectomy for diverticular disease, potentially influencing treatment selection in favor of non-operative strategies [29].

Additionally, elevated CRP levels were associated with radiological drainage use, suggesting that inflammatory markers may influence the decision to add drainage to conservative approaches, as confirmed by previous studies [30].

Contrary to expectations, age, sex, WBC, and the treatment period (pre- or during COVID-19) did not significantly impact treatment selection. These results suggest that objective clinical indicators, rather than demographic characteristics or pandemic-related factors, primarily guide therapeutic choices in this patient population.

This study benefits from a comprehensive and well-structured dataset spanning two equivalent timeframes before and during the COVID-19 pandemic. The inclusion of various parameters, ranging from patient demographics and disease severity to management strategies and follow-up outcomes, provides a holistic view of how the pandemic influenced the management of ACD. The detailed analysis highlights changes in admission and treatment patterns and identifies key factors, such as inflammatory markers, that reflect patient care during this period.

Despite its strengths, this study is limited by its single-center design, which may restrict the generalizability of its findings to other healthcare systems with varying resources and pandemic responses. The retrospective nature of the research introduces potential biases, including reliance on the accuracy and completeness of electronic medical records, as well as the risk of selection bias. Moreover, unmeasured confounding variables may have influenced treatment decisions and outcomes, limiting the ability to draw definitive causal conclusions. While the overall sample size is acceptable, it may still limit the robustness of certain statistical comparisons. Lastly, the relatively short follow-up period of 90 days may not have fully captured long-term outcomes, such as recurrence rates or late complications, thereby narrowing the scope of the findings.

## Conclusions

This study reveals a marked decline in hospital admissions for ACD during the COVID-19 pandemic, despite comparable baseline characteristics and disease severity between the pre-COVID and COVID periods. Although the overall use of NOM remained high, there was a noticeable shift toward a greater use of radiological drainage and a modest increase in surgical interventions during the pandemic.

The analysis showed that treatment choices were not influenced by the period of admission but were determined by objective clinical parameters, specifically, higher Hinchey grade and elevated inflammatory markers such as CRP. Moreover, patients with a higher BMI were more frequently managed conservatively, possibly due to considerations of perioperative risk.

These findings underscore the importance of flexible yet consistent treatment strategies that prioritize individualized, high-quality care, even during a challenging period such as the COVID-19 pandemic.
